# Advancing social care integration in health systems with community health workers: an implementation evaluation based in Bronx, New York

**DOI:** 10.1186/s12875-024-02376-7

**Published:** 2024-04-27

**Authors:** Kevin P. Fiori, Samantha Levano, Jessica Haughton, Renee Whiskey-LaLanne, Andrew Telzak, Hemen Muleta, Kavita Vani, Earle C. Chambers, Andrew Racine

**Affiliations:** 1 Department of Pediatrics, Albert Einstein College of Medicine, 1300 Morris Park Avenue, Bronx, NY 10461 USA; 2 Department of Family and Social Medicine, Albert Einstein College of Medicine, 1300 Morris Park Avenue, Bronx, NY 10461 USA; 3 Department of Obstetrics & Gynecology and Women’s Health, Albert Einstein College of Medicine, 1300 Morris Park Avenue, Bronx, NY 10461 USA

**Keywords:** Health related social needs, Community health workers, Health equity, Primary health care, Social determinants of health, Social care integration

## Abstract

**Background:**

In recent years, health systems have expanded the focus on health equity to include health-related social needs (HRSNs) screening. Community health workers (CHWs) are positioned to address HRSNs by serving as linkages between health systems, social services, and the community. This study describes a health system’s 12-month experience integrating CHWs to navigate HRSNs among primary care patients in Bronx County, NY.

**Methods:**

We organized process and outcome measures using the RE-AIM (Reach, Effectiveness, Adoption, Implementation, Maintenance) implementation framework domains to evaluate a CHW intervention of the Community Health Worker Institute (CHWI). We used descriptive and inferential statistics to assess RE-AIM outcomes and socio-demographic characteristics of patients who self-reported at least 1 HRSN and were referred to and contacted by CHWs between October 2022 and September 2023.

**Results:**

There were 4,420 patients who self-reported HRSNs in the standardized screening tool between October 2022 and September 2023. Of these patients, 1,245 were referred to a CHW who completed the first outreach attempt during the study period. An additional 1,559 patients self-reported HRSNs directly to a clinician or CHW without being screened and were referred to and contacted by a CHW. Of the 2,804 total patients referred, 1,939 (69.2%) were successfully contacted and consented to work with a CHW for HRSN navigation. Overall, 78.1% (*n* = 1,515) of patients reported receiving social services. Adoption of the CHW clinician champion varied by clinical team (median 22.2%; IQR 13.3–39.0%); however, there was no difference in referral rates between those with and without a clinician champion (*p* = 0.50). Implementation of CHW referrals via an electronic referral order appeared successful (73.2%) and timely (median 11 days; IQR 2–26 days) compared to standard CHWI practices. Median annual cost per household per CHW for the intervention was determined to be $184.02 (IQR $134.72 – $202.12).

**Conclusions:**

We observed a significant proportion of patients reporting successful receipt of social services following engagement with an integrated CHW model. There are additional implementation factors that require further inquiry and research to understand barriers and enabling factors to integrate CHWs within clinical teams.

**Supplementary Information:**

The online version contains supplementary material available at 10.1186/s12875-024-02376-7.

## Background

Addressing the ways in which social needs influence health outcomes is critical to improving the health, well-being, and quality of life of communities and individuals. Health-related social needs (HRSNs) are defined as the self-reported individual experiences of social risk factors at a particular moment in time and may include needs such as housing stability, food security, or transportation access [1]. HRSNs are distinct from the broader, structural social determinants of health (SDoH), which are defined by the World Health Organization as “the conditions in which people are born, grow, work, live, and age, and the wider set of forces and systems shaping the conditions of daily life” [2]. In recent years, health systems have expanded their focus from access and quality of health care to include screening for and addressing HRSNs, which can influence more than 50% of a patient’s health [3]. Increasing clinical teams’ awareness of HRSNs is an important first step in addressing health disparities, as outlined by the National Academies of Sciences, Engineering, and Medicine (NASEM) [1]. Health systems have primarily integrated awareness activities by screening patients for HSRNs [4].

There remain clear gaps in how to best provide assistance once patients report HRSNs [5]. Health systems have demonstrated variability in linking patients with HRSNs to appropriate resources and social service providers, also known as “social prescribing” [6]. These decisions have recently shifted from the health system to regulatory agencies, which have identified HRSN screening and referral as an emerging priority. The Centers for Medicare & Medicaid Services (CMS) released new health equity measures, which mandate reporting and screening of key SDoH domains in inpatient settings in 2024 [7]. Meanwhile, the Joint Commission now requires health systems to screen patients for HRSNs as well as to address the HRSNs identified through a defined action plan [8].

Social prescribing activities may include referrals to onsite resources or community-based organizations, and involve nurses, social workers, student volunteers, or community health workers [6]. Community health workers (CHWs) are frontline public health workers with deep understanding of, and trust within local communities [9]. CHWs often serve as intermediaries between health care institutions, social services, and the community because they are uniquely positioned to facilitate access to resources, improve cultural competence of care delivery, strengthen patient self-sufficiency, and support communities in addressing the underlying causes of health disparities [10].

There is evidence supporting the effectiveness of CHW interventions in the US in improving patient care, reducing cost of care, and advancing health equity [11]; however, few studies have evaluated the impact of CHWs on HRSNs in real world practice. Previous randomized control trials (RCTs) examining CHW social prescribing interventions have reported reduced hospitalizations [12], improved health-related quality of life [13], improved access to primary care after hospitalization [14], and improved child health status [15] when compared to usual care. Additional CHW interventions have focused evaluations on the resolution of social needs [15] and connection to essential social services [16–18]. There is a demonstrated gap in understanding how a large health system can successfully integrate an enterprise wide CHW program into clinical practice. This study’s objective was to describe and evaluate a health system’s 12-month experience integrating CHWs within clinical teams to address HRSNs among primary care patients in a large, multi-cultural, resource-constrained system in Bronx County, NY.

## Methods

### Intervention

The Community Health Worker Institute (CHWI) at Montefiore Medical Center strives to improve health equity by optimizing the integration of CHWs within clinical care teams using a learning health system approach [19]. CHWs serve as a bridge between social and clinical care by addressing HRSNs and improving access to healthcare for disadvantaged populations. The CHWI centralizes recruitment, training, continuing professional development, and deployment of CHWs embedded within the health system.

Montefiore Medical Center primarily serves patients from Bronx County, New York, which is home to more than 1.3 million people [20]. It is one of the most diverse counties in the US, with 56.6% of its residents identifying as Hispanic and 44.3% as non-Hispanic Black. Bronx County consistently ranks among the least healthy counties in New York State in measures of health factors and health outcomes [3]. In spite of these challenges, the Bronx also has many assets and resources including access to higher education, healthcare facilities, open spaces, and community- and faith-based organizations [21].

The CHWI builds on an initial pilot, the Community Linkage to Care (CLC) program, which modeled standardized HRSN screening and CHW referral support in our health system in 2017 [22]. As part of the CLC program, the health system implemented a 10-item HRSN screening tool adapted from the Health Leads Toolkit (Supplemental Fig. 1) [23]. In 2022, the health system centralized CHW operations within the CHWI structure, representing a novel and enhanced investment towards CHW integration within the health system.

The CHWI was developed in partnership with key stakeholders including community-based organizations, CHW workforce pipeline programs, clinical teams, existing CHWs, and CHW networks. Leadership and management of the CHWI include former CHWs, which integrates their perspective into operations and implementation. The CHWI has also taken the steps to establish a Community Advisory Board (CAB) to represent the interests of patient representatives, local community leaders, and partners from the social service providers’ network. We utilize these established relationships to ensure the CHWI is meeting the needs of our catchment population, involve the community in CHW recruitment, and co-design new programs and proposals.

The CHWI has collaborated with multi-sector partners to recruit socially connected community residents who share the lived experiences of our patients. These CHWs are full-time employees with competitive salaries and robust union benefits packages who receive training, supervision, and mentorship immediately upon hire. Implementation of the CHWI includes a training plan composed of standards and guidelines for hiring and deployment, a comprehensive training curriculum, an accredited apprenticeship program with a local community college, a detailed scope of practice, and a peer-management structure. The supportive supervision structure is a key component of the CHWI, meeting with individual CHWs weekly and in groups monthly to review successes and challenges, provide mentorship, share bi-directional feedback, support professional development, and foster retention.

The CHWs employed by the CHWI provide social service navigation and referral support to patients with HRSNs within their assigned clinical practices. If a patient self-reports at least one HRSN during a clinical encounter, clinicians discuss the need or review the results of the screening tool, if screened, with the patient and their family members and ask whether they are interested in receiving assistance with their HRSN(s). If assistance is requested, clinicians send an electronic referral order within the health system’s electronic health record (EHR) to facilitate connection to CHWs. If assistance is requested but a CHW is not available, clinical teams utilize the health system’s EHR-supported social service directory to find available resources to refer patients to in their local community.

After receiving an electronic referral order from a clinician, CHWs contact the patient and/or their family member to collect household and demographic information, re-screen for HRSNs, confirm consent for assistance, and begin HRSN navigation. During HRSN navigation encounters, CHWs screen patients for eligibility criteria for public benefits programs, complete or assist with benefits applications, refer patients to community-based organizations, and provide direct benefits on site, when available. After CHWs complete in-person or telephone navigation encounters with patients, they conduct outreach with other clinical and community-based care team members and document connection to social services and resolution of HRSNs. CHWs conduct ongoing follow-up with each patient until their HRSN is resolved, no more progress can be made, the patient is disconnected, or the patient is no longer interested in receiving assistance from the CHW.

In prior work, we have demonstrated the successful reach and adoption of the HRSN screening arm of the previous CLC program [24]. This study aims to evaluate the centralized CHW referral component of the enhanced CHWI program.

### Study design & data sources

This retrospective, cross-sectional study utilized data collected and managed through Research Electronic Data Capture (REDCap) tools to assess the integration of CHWs across the health system. REDCap is a secure, web-based software platform hosted at Albert Einstein College of Medicine, which is designed to support data capture for research studies [25, 26]. The CHWI REDCap database is a tool designed for CHWs to routinely collect data on patient demographics, referral information, outreach encounters, social need services provided, and key program outcomes. This database was internally developed and has been continually updated by the CHWI team as part of our learning health system approach. Since the launch of CHWI, we have added new services offered in the community, integrated REDCap data access groups for CHW training, automated data entry processes for follow-up encounters, linked family members through unique record numbers, and streamlined reports to track patients with ongoing follow-up needed.

Additional data was extracted from the EHR to identify patients eligible to be referred to CHWs through HRSN screening and electronic referral order databases. All EHR data was extracted using Microsoft SQL Server, version 18, to query data from the Epic Electronic Health Record Data Warehouse.

### Study population

Patients were eligible for inclusion in the analysis if they were referred to a CHW for HRSN service navigation and support within participating clinical teams and the first outreach was attempted by a CHW between October 2022 and September 2023. This time period reflects when the CHWI deployed its first cohort of CHWs within the system. Patients were initially referred to a CHW if they met the following eligibility criteria: (1) self-reported HRSNs by a standardized screening tool and requested assistance from a clinician, (2) self-reported HRSNs during the clinician visit and requested assistance from a clinician without being screened, or (3) self-reported HRSNs and requested assistance directly from the CHW, if on-site, without being screened. If the patient requested assistance directly from the CHW, the CHW would meet with the patient, contact the patient’s primary clinician, and request that the clinician retroactively submit an electronic referral order. We focused on an initial 12-month period to account for seasonality with additional follow-up outreach and outcomes for the patient after September 2023 being excluded from the analysis.

### Study measures

We organized both process and outcome measures using the RE-AIM (Reach, Effectiveness, Adoption, Implementation, Maintenance) implementation framework domains (Table 1) [27]. This framework facilitated and organized assessments of implementation with the study period. We utilized data from five ambulatory pediatric and five ambulatory internal medicine clinical teams that were actively participating in this initiative during the study period. All process and outcome measures were focused on these ten clinical teams.

**Table 1:** RE-AIM community health worker institute (CHWI) program components, measures, and key data sources

Components	Measures	Key Data Sources
**Reach**	R1: % of eligible patients screened positive for HRSNs who were referred to a CHW R2: total number of eligible patients referred to a CHW	• EHR HRSN Screening Database • CHWI REDCap Database
**Effectiveness**	E1. % of eligible patients connected to at least one social service E2. % of eligible patients who self-reported resolution of progress on at least on HRSN	• CHWI REDCap Database
**Adoption**	A1. % of eligible patients screened positive for HRSNs who were referred to a CHW by clinical team A2. % of clinical teams with a clinical champion present during the study period	• EHR HRSN Screening Database • CHWI REDCap Database • Clinician Champion Tracking Sheet
**Implementation**	I1. % of referrals received via electronic referral order entered into CHWI database I2. median time, in days, between the electronic referral order date and first CHW contact date	• EHR Electronic Referral Order Database • CHWI REDCap Database
**Maintenance**	M1. median monthly cost per household per CHW	• Health System Operations Budget

### Reach

We defined reach as the (1) proportion of patients with self-reported HRSNs in the screening tool who were referred to and contacted by a CHW (i.e., eligibility criteria 1 only) and (2) the total number of patients who self-reported HRSNs by any means and were referred to and contacted by a CHW (i.e., eligibility criteria 1, 2, and 3). Patients were eligible to be referred to a CHW if they self-reported HRSNs with or without a screener; therefore, the second reach measure estimates the absolute coverage of the CHWI program. We utilized the first reach measure to estimate the potential drop-off between initial self-report of HRSNs and first attempted contact by the CHW because the screening tool provides the only standardized documentation of patients with self-reported HRSNs who may decline assistance from a CHW prior to the clinician electronic referral order.

To determine the proportion of patients eligible to be referred and contacted, we matched patients with self-reported HRSNs in the screening tool from the clinical teams of interest between October 2022 and September 2023 with patients contacted by CHWs from those clinical teams during the same time period using unique identifiers. For the second reach measure, we calculated the total number of patients contacted by CHWs from these clinical teams during the study period, since not all patients referred may have been screened using the standardized tool.

For the total patients referred and contacted, we further described their referral status based on their stage in the initial contact and consent process. We reported socio-demographic covariates, including age at referral, gender, race/ethnicity, and preferred spoken language, which were collected and documented by CHWs in the CHWI REDCap Database.

### Effectiveness

We defined our primary effectiveness measure as the proportion of patients assisted with HRSNs for which the CHW connected (i.e., received) or equipped the patient with the tools to connect to at least one social service on their own. These outcomes are both considered successful because the CHW completed all necessary steps, per program workflows, to connect the patient to available services. We also defined a secondary effectiveness measure as the proportion of patients connected to at least one social service who resolved or made progress on at least one HRSN. This secondary measure was self-reported by the patient and only answered by those connected to at least one social service, not those equipped to complete connection on their own. These measures were based on internally standardized definitions for all social needs and services (Supplemental Table 1).

### Adoption

Adoption of the intervention was measured according to two measures, (1) the proportion patients with self-reported HRSNs in the screening tool who were referred to and contacted by a CHW by each clinical team and (2) the proportion of clinical teams establishing a clinician champion as recommended by the intervention. Clinician champions were defined as “full-time clinicians based at practice who serve as a clinical contact, mentor, and/or coach to support CHW team integration and lead performance improvement initiatives” and have been previously demonstrated to increase screening rates in our health system [22, 28]. In other studies, clinician champions have demonstrated their ability to influence the behavior of other clinicians, challenge institutional norms, leverage professional relationships, cultivate a learning environment, and optimize existing workflows [29, 30]. This role was implemented alongside the CHWI peer management structure so that CHWs would have an advocate within the clinical team to facilitate the HRSN screening and referral process. We used two sample T-tests to estimate whether the proportion of patients referred and contacted differed for clinical teams with and without clinician champions present.

### Implementation

Implementation measures included fidelity measures of the extent to which clinical teams and CHWs adhered to recommended, established workflows. These measures included (1) the proportion of patients with electronic referral orders who were referred to and contacted by a CHW, and (2) the median time, in days, between the clinician’s electronic referral order and the first CHW outreach attempt. It was an operational expectation by the CHWI for CHWs to contact patients within 7 days of the electronic referral order by a clinician. The CHWI also encouraged clinicians to complete a warm handoff, or transfer of care, with the CHW after the on-site clinical visit as part of their standardized workflow. Due to potential delays in the documentation of the electronic referral orders in the EHR, we confirmed the utilization of a "warm handoff" in the CHWI REDCap Database and assigned the time between the order date and first outreach as 0 days for these cases. We excluded all other observations when the first CHW outreach attempt was dated prior to the electronic referral order date.

We did not assess time between the HRSN screen and electronic referral order since this is dependent solely on the clinician placing an order. To determine the proportion of patients with electronic referral orders contacted, we matched the EHR electronic referral order and CHWI REDCap databases using unique identifiers.

### Maintenance

Maintenance was defined as the cost to sustain the CHWI intervention over time given the need to establish an estimated ongoing annual cost per beneficiary. This was measured by estimating the median annual cost to the health system per patient referred to a CHW. We first determined the annual patient count for each CHW and annual cost per CHW based on standardized CHWI salary estimates and time contributed by each CHW during the annualized study period. We then calculated the annual cost to the health system per patient for each CHW by dividing the annual patient count by the annual cost per CHW. Next, we calculated the weighted median annual cost to the health system per patient across the study period, with weights based on the proportion of months that the CHW participated in the intervention. We excluded observations for CHWs if they contributed partial data due to mid-month deployment or departure, provided only supplemental coverage to the clinical teams of interest, or demonstrated abnormal data due to performance concerns.

### Analysis

We used both descriptive and inferential statistics to summarize socio-demographic characteristics of patients referred and RE-AIM process and outcome measures. All descriptive and inferential statistics were conducted in SAS version 9.4. Weighted estimates for the Maintenance measure were calculated using PROC MEANS in SAS. All research was approved by the Albert Einstein College of Medicine Institutional Review Board (2017–8434).

## Results

### Reach

Between October 2022 and September 2023, 25,996 unique patients were screened for HRSNs within the ten participating clinical teams using the screening tool, with 4,420 (17.0%) reporting at least one unmet HRSN. Of the patients who self-reported HRSNs in the screening tool, 1,782 were referred by clinicians via electronic referral order and 1,245 were successfully referred to and contacted by (i.e., completed the first outreach attempt) CHWs integrated within these clinical teams during the study period. An additional 1,559 patients self-reported HRSNs directly to a clinician (i.e., eligibility criteria 2) or CHW (i.e., eligibility criteria 3) and were referred to CHWs who attempted the first outreach.

We summarized the socio-demographic characteristics of the total referred population (*n* = 2,804) (Table 2). Most patients referred were between 0 and 5 years of age (27.7%) followed by those between 30 and 64 years (23.9%). There were more women (55.7%) than men referred (43.8%). Referred patients most identified as Hispanic (42.4%) or Non-Hispanic Black (28.2%), with many patients declining to report their race or ethnicity (22.0%). Most patients preferred English as their primary language (74.3%) followed by Spanish (21.6%). There were 2,993 total HRSNs reported with the most reported HRSNs identified as housing security (24.7%), food security, (20.3%) and financial benefits (16.9%), respectively (Table 3).

**Table 2:** Descriptive Characteristics of Patients with Self-Reported Health-Related Social Needs Referred to Community Health Worker Institute, October 2022- September 2023

Measures	Number of Patients Referred (n, %)
**Total Patients**	2,804 (100.0%)
**Age, in years**	
0–5	777 (27.7%)
6–11	487 (17.4%)
12–19	333 (11.9%)
20–24	71 (2.5%)
25–29	69 (2.5%)
30–64	670 (23.9%)
65+	397 (14.2%)
**Gender**	
Man	1,227 (43.8%)
Woman	1,562 (55.7%)
Transgender Man	1 (0.0%)
Transgender Woman	3 (0.1%)
Gender Non-Conforming	1 (0.0%)
Other Gender	1 (0.0%)
Declined to Report	8 (0.3%)
Missing	1 (0.0%)
**Race and Ethnicity**	
Non-Hispanic White	59 (2.1%)
Hispanic	1189 (42.4%)
Non-Hispanic Black	792 (28.2%)
Non-Hispanic Asian / Pacific Islander	40 (1.4%)
Non-Hispanic American Indian / Alaskan Native	11 (0.4%)
Other	56 (2.0%)
Declined to Report	618 (22.0%)
Missing	39 (1.4%)
**Preferred Spoken Language**	
English	2,084 (74.3%)
Spanish	605 (21.6%)
Bilingual, Spanish or English	31 (1.1%)
Other Language	78 (2.8%)
Missing	6 (0.2%)

**Table 3:** Self-reported health-related social needs (HRSNs) of patients referred to the community health worker institute, october 2022-september 2023

Measures	Total HRSNs Identified (n, %)
**Total HRSNs**	2,993 (100.0%)
Housing Security	739 (24.7%)
Housing Quality	313 (10.5%)
Employment	98 (3.3%)
Financial Benefits	505 (16.9%)
Food Security	607 (20.3%)
Care Coordination & Navigation	176 (5.9%)
Referral to Health Homes Program	0 (0.0%)
Legal Services	168 (5.6%)
Youth & Family Services	312 (10.4%)
Referral to Primary Care Provider	7 (0.2%)
Other Need	68 (2.3%)

There were 2,804 total patients referred from these clinical teams with the first outreach attempt completed by CHWs between October 2022 and September 2023. These patients were referred to a total of 28 full-time employed CHWs, who each contributed a median of 3.0 months (IQR 2.0–6.5 months) during the 12-month study period. We collected demographic data for 23 of the 28 CHWs included in the analysis. Most of the CHWs identified as Black or African American (65.2%) followed by other race (13.0%). Almost half (47.8%) of the CHWs identified as Hispanic or Latino. Most CHWs were between the ages of 25 to 34 years (65.2%) followed by those between 35 and 44 years (13.0%). Almost all CHWs identified as female (95.7%).

CHWs completed 12,376 total outreach encounters with these patients, with 84.9% of outreach attempts completed by phone, 7.7% in person, and the remaining by alternative or unknown methods. This includes 7,509 HRSN navigation encounters, with most navigation encounters (52.3%) reported as 15 min or less in duration. CHWs conducted HRSN navigation with patients and their families over a median of 31 days (IQR 8–70 days) across the study period.

**Fig. 1:**
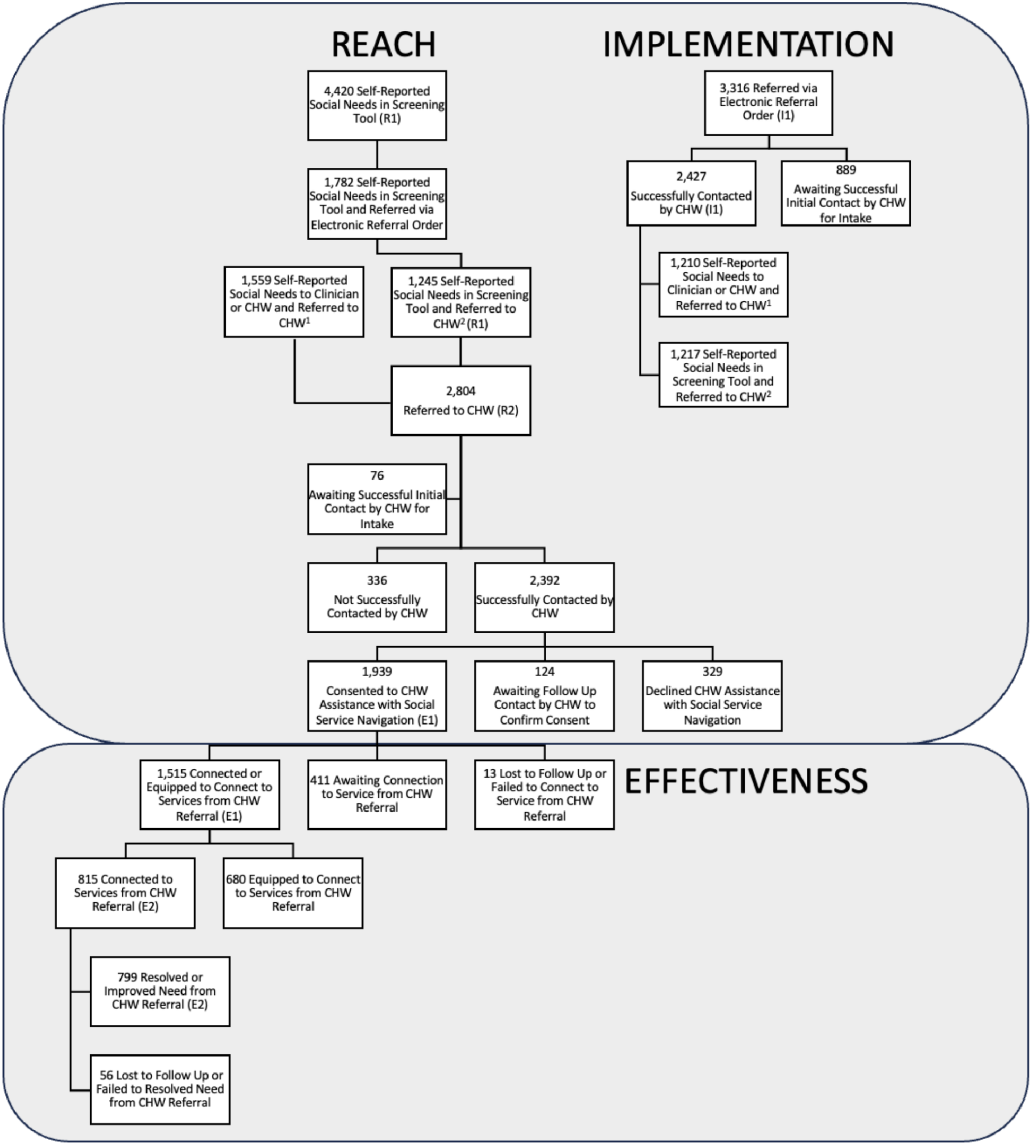
Reach and effectiveness of the community health worker institute in connecting patients with self-reported health-related social needs to services, October 2022- September 2023. ^1^ 1,210 of 1,559 patients identified through direct report of HRSNs to a clinician or CHW were referred via electronic referral order during the study period. ^2^ 1,217 of 1,245 patients identified through the screening tool were referred via electronic referral order during the study period

Of the patients referred with attempted contacted, 1,939 (69.2%) were successfully contacted and consented to work with a CHW to address self-reported HRSNs. An additional 124 (4.4%) patients were successfully contacted but have yet to consent to CHW assistance, 329 (11.7%) were successfully contacted but declined CHW assistance, 336 (12.0%) were disconnected after three or more unsuccessful initial outreach attempts, and 76 (2.7%) patients were still awaiting successful initial contact by a CHW (i.e., have not three or more initial outreach attempts) at the time of data analysis (Fig. 1).

### Effectiveness

We measured CHW effectiveness through patient reported connection to social services. Overall, 1,515 (78.1%) of the 1,939 patients were connected (i.e., received) (*n* = 835) or equipped to connect (*n* = 680) to social services (Supplemental Table 1). Meanwhile, 13 (0.7%) patients failed to connect to services or were lost to follow-up before services could be confirmed. The remaining 411 (21.2%) patients are still actively working with a CHW at the time of study end period (Fig. 1).

Of the 815 patients who were connected to a social service (i.e., received only), 779 (93.3%) reported that their HRSN was improved or fully resolved. Approximately 56 (6.7%) patients reported that they failed to make progress on their HRSN or were not able to document progress because they were disconnected from care.

### Adoption

In our health system, every clinical team approached during the study period integrated CHWs into their team for HSRN navigation. Of the ten clinical teams, adoption of the referral component of the intervention varied with a range of 6.4–72.4% of eligible patients referred to a CHW, with a median referral rate of 27.8% (IQR 14.9–44.6%) (Table 4). We also measured adoption of a key aspect of the intervention, the adoption of a clinician champion within the team. Approximately 80% (*n* = 8) of clinical teams had recruited or retained a clinician champion during the study period to collaborate with the CHWI program team, educate other clinicians on the referral process, and discuss barriers and facilitators to implementation. There was no difference in the average rate of referral usage between clinical teams with (80%) and without (20%) a clinician champion present (*p* = 0.50).

**Table 4:** Adoption of the Community Health Worker Institute Referral Program by Participating Clinical Teams, October 2022- September 2023

Clinical Team	Clinician Champion Present	Number of Patients with Self-Reported HRSNs in Screening Tool	Number of Patients Referred to and Contacted by CHW	Percent of Patients Referred to and Contacted by CHWs
Clinical Team 1	Yes	188	7	3.7%
Clinical Team 2	No	285	30	10.5%
Clinical Team 3	Yes	195	26	13.3%
Clinical Team 4	Yes	753	104	13.8%
Clinical Team 5	Yes	581	115	19.8%
Clinical Team 6	No	102	25	24.5%
Clinical Team 7	Yes	844	240	28.4%
Clinical Team 8	Yes	528	206	39.0%
Clinical Team 9	Yes	596	253	42.4%
Clinical Team 10	Yes	348	239	68.7%
TOTAL	80%	4,420	1,245	Median 22.2% (IQR 13.3–39.0%)

### Implementation

There were 3,316 patients with electronic referral orders sent by clinicians, after self-reporting HRSNs in the screening tool or directly to a clinician or CHW, between October 2022 and September 2023. CHWs completed the first outreach attempt for 2,427 (73.2%) of these patients, who are included in our study sample, with the remaining 889 patients (26.8%) still awaiting initial outreach by a CHW at the time of data analysis. Of the 2,427 patients contacted, 1,210 were initially identified through direct report of HRSNs to a clinician or CHW while 1,217 were identified through the screening tool (Fig. 1). There were 377 patients, of the total 2,804 referred patients in the study sample, who were excluded in this assessment because their electronic referral orders were sent outside of the study period.

We measured the time between the patient’s electronic referral order and first outreach attempt by the CHW to better understand implementation of the intervention by CHWs. The median time for CHWs to first contact the patient was 11 days (IQR 2–26 days) after the electronic referral order, compared to standard CHWI expectation of 7 days. There were 273 patients whose electronic referral order and first outreach attempt were reassigned to the same day because their clinicians completed a warm handoff with the CHW, as confirmed in the CHWI REDCap Database. There were 392 patients with electronic referral orders sent after the CHW’s first outreach attempt that were excluded from this study.

### Maintenance

There were 12 CHWs included in our assessment of median annual cost to the health system per patient to sustain the CHWI intervention. After applying analytic weights, calculated based on the number of months contributed by each CHW to the intervention, we determined that the median annual cost per patient was $184.02 (IQR $134.72 – $202.12).

## Discussion

The CHWI reached over 2,800 patients in its initial 12-month roll-out period and was effective in linking nearly 80% of patients assisted to resources. Adoption of the CHWI intervention components varied by participating clinical team, with no difference in referral rates between clinical teams with and without a clinician champion present. Implementation of CHW referrals via the electronic referral order from clinicians and initial contact by CHWs was overall successful and timely. Maintaining CHWs in our health system will require more sustainable funding as adoption of the program expands; however, the median cost per patient provides a baseline cost estimate to prepare for future reimbursement and value-based payment models.

This evaluation expands and adds to existing knowledge of assessing real-world implementation of social prescribing interventions. In 2022, the RE-AIM framework was utilized to evaluate a similar ambulatory social care program, which reached 34% of patients who screened positive for HRSNs and connected 75% of participants to social services [31]. This program is comparable in scale to the catchment population of the CHWI but limited to pediatric settings. Additional programs focused on CHWs and HRSNs have demonstrated varying estimates of reach but at smaller scale [16] and within different settings [32].

Preliminary results from the largest HRSN screening and referral program in the US, the American Health Communities (AHC) Model, have demonstrated a much higher acceptance rate, reaching closer to 80% of eligible participants [33]. This model, however, has also suggested that HRSN navigation alone is not effective in increasing connection to social services or resolving social needs [33]. There were significant barriers noted by beneficiaries and service providers in accessing or confirming connection to services that likely contributed to the effectiveness of HRSN navigation. Additionally, services accessed were not always enough to meet the needs of the beneficiaries. Recommendations from this study include assessing and investing in local community service provider capacity and exploring additional mechanisms, other than addressing HRSNs, through which navigation programs may contribute to health outcomes.

Our experience has suggested that effective integration of CHWs within health systems is challenging as the role is often novel with ambiguous roles and scope. Prior to engagement with CHWs, clinical teams may struggle with understanding the CHW role or experience conflict while transitioning from traditional care models [34]. In a qualitative study in Chicago, higher levels of CHW integration were found in clinical teams with greater alignment in CHW purpose and value perspectives across administrators, clinicians, and CHWs [35]. Additional studies indicated that communication channels, internal and external trainings, community expertise, and opportunities for CHW connection were most valuable to CHW integration [36, 37]. Despite the finding that clinician champions were not associated with adoption of CHW referrals in our study, these staff members have increased access and opportunity to educate other clinicians on the purpose and value of the CHWI referral program. Clinician and administrative champions were identified as key facilitators to CHW integration in a study in Minnesota, along with a culture of innovation, prioritization of nonmedical determinants of health, and sustainable reimbursement strategies [38].

Few studies have successfully demonstrated the direct economic impact of CHW interventions. In a recent analysis as part of a RCT in Pennsylvania, the CHW program demonstrated an annual return of $2.47 for every dollar invested annually by Medicaid, which further incentivizes state Medicaid programs and health systems to invest in CHW programs to improve health outcomes, address HRSNs, and lower costs [39]. Several states have started utilizing CHW services to address population health needs and have authorized their payments through Medicaid programs [40]. In 2023, New York State (NYS) Medicaid announced that health care providers would be reimbursed for CHW services. This funding was initially limited to pregnant and postpartum individuals but will be expanded to children and adults with HRSNs in 2024, which will directly impact the maintenance of the CHWI [41].

In NYS, CHWs will be reimbursed at a rate of $35.00 per Medicaid member for individual education and training sessions, with 12 annual sessions allowed for adults and 24 for children [42]. The CHWI developed its maintenance measure based on the number of patients served per year rather than the number of sessions administered, given that it does not currently limit the number of sessions per patient. Additionally, the CHWI does not administer group navigation sessions, for which Medicaid also offers rates of reimbursement [42]. These are factors that the CHWI model may need to consider adapting or may need to advocate against as non-practical elements of the Medicaid-reimbursement model. There are additional opportunities for sustainable funding in NYS to expand the CHWI, including the Sect. 1115 waiver and shift from Fee for Service towards Value Based Payment models [40]. As new models are released and updated, it is important that we continue to compare our baseline cost estimates to determine the need for future adaptation and advocacy work.

### Limitations

This study has several limitations to address. First, patients with self-reported HRSNs identified in the screening tool are not representative of all active patients with HRSNs in the health system. Although universal screening is recommended, clinical teams have the discretion to screen their patient population based on pre-defined intervals or eligibility criteria. Additionally, patients who do complete the screen may not self-report HRSNs due to a lack of trust in the health care system [43]. As previously mentioned, patients may also self-report HRSNs directly to the clinician or CHW without being screened; however, there is currently no documentation of these patients unless the patient requests assistance and the clinician sends an electronic referral order or the CHW completes the first outreach attempt.

There are also limitations in documenting patients who self-report HRSNs in the screening tool but do not request assistance with HRSNs. We are able to document rates of acceptance for these patients; however, this measure is not widely utilized with significant missing data observed [44]. We also do not have data available for patients who requested assistance and were referred to the EHR-supported social service directory when a CHW was not available.

There are additional limitations related to the workflow between clinicians and CHWs. The health system recommends, but does not require, that clinicians screen patients for HRSNs prior to completing an electronic referral order. Therefore, not all patients referred will be identified as eligible in the EHR screening database. Additionally, there are challenges in matching the EHR database with the REDCap CHWI database due to lag times between screening, electronic referral order, and first CHW contact dates.

Finally, data collection for HRSN screening and CHW referrals were conducted by non-research staff as part of routine service delivery. Although data entry safeguards were utilized and data were regularly reviewed by investigators, there is potential for misclassification bias and data entry errors. Our process and outcome measures have specific limitations given the nature of social service referrals. We define success according to the CHWs ability to facilitate connection to services rather than the patient’s actual receipt of benefits, which is challenged by the barriers and limitations of the social service industry. This definition may overestimate the “true” magnitude of our interventions effect on HRSNs. On the other hand, we define effectiveness as unsuccessful when patients are disconnected from CHWs, which may underestimate that same effect.

## Conclusions

We conducted an implementation evaluation of a real-world CHW intervention aimed at connecting patients with HRSNs to social services. Despite significant proportions of patients both connecting to social services and reporting progress or resolution of HRSNs after receipt of CHW assistance, there is more optimization work ahead. We need to better understand why a meaningful proportion of patients with HRSNs either decline assistance or are not successfully connected to CHW navigation services. Furthermore, we need more rigorous costing assessments to understand how health systems can sustain this workforce. The shift of health systems to improve social care integration portends improved health outcomes and reduced health disparities, but there is more research and learning required to achieve these goals.

### Electronic supplementary material

Below is the link to the electronic supplementary material.

Supplementary Material 1

## Data Availability

The de-identified datasets used and/or analyzed during the current study are available from the corresponding author on reasonable request.

## Abbreviations

HRSN: health-related social nee
SDoH: social determinants of health
CHW: community health worker
RCT: randomized control trial
CHWI: Community Health Worker Institute
CLC: Community Linkage to Care
HER: electronic health record
REDCap: Research Electronic Data Capture
