# Supplementary material for: Advancing social care integration in health systems with community health workers: an implementation evaluation based in Bronx, New York
Source: BMC Prim Care. 2024 Apr 27;25:140. doi: 10.1186/s12875-024-02376-7 (PMC11055265; doi:10.1186/s12875-024-02376-7)
Supplement: Supplementary file 1 — Supplementary Material 1 [file 12875_2024_2376_MOESM1_ESM.docx]

**Supplemental Fig. 1. Health-Related Social Needs Screening Tool, December 2019 – October 2023**


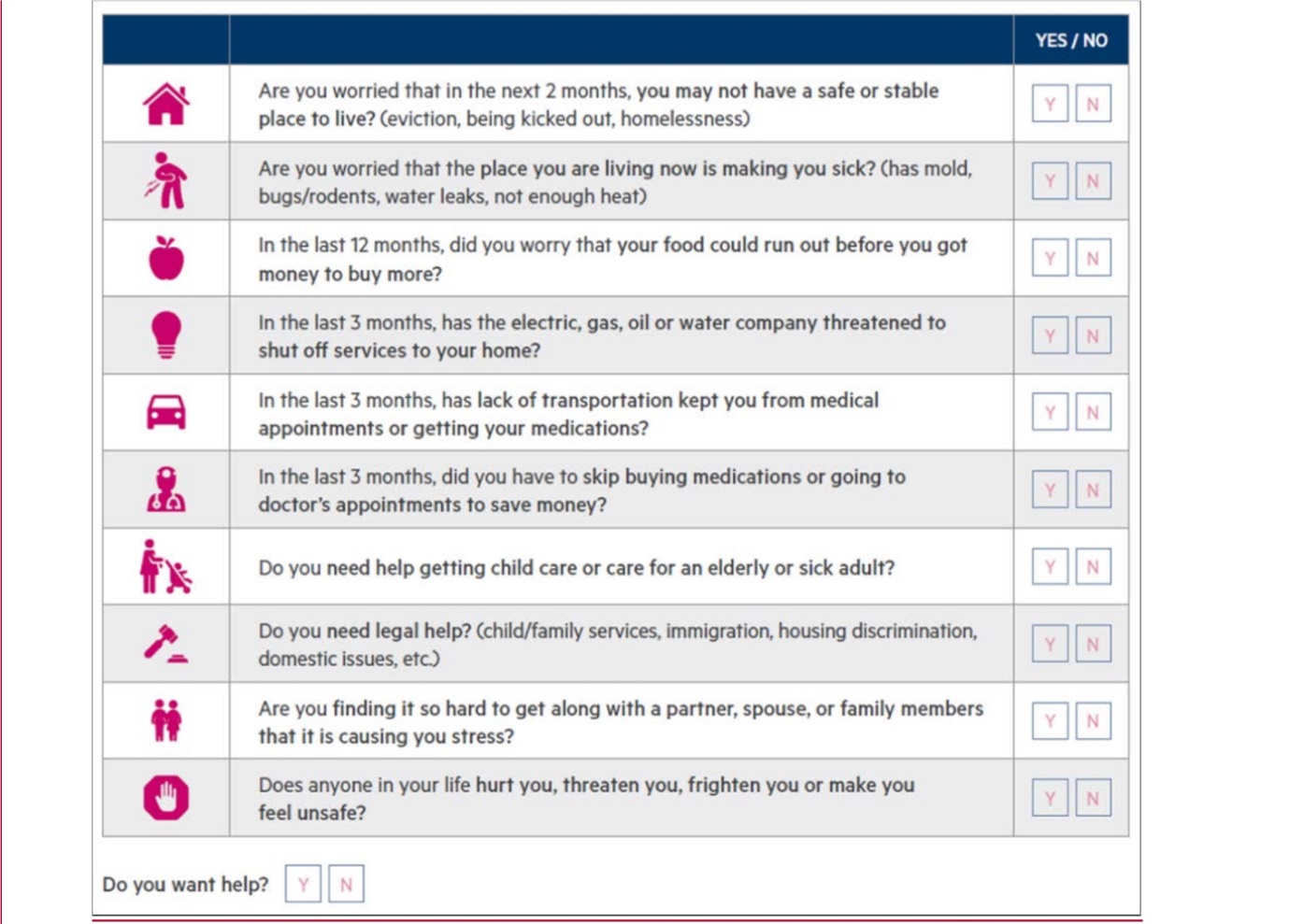


| **Supplemental Table 1. Definitions of Primary and Secondary CHWI Effectiveness Measures** | |
| --- | --- |
| **Measure** | **Definition** |
| **Primary Effectiveness Measure:** proportion of patients assisted with HRSNs for which the CHW completed all necessary steps, within their ability, to connect the patient to at least one social service | |
| **Received** (i.e., Successful) | Referral has been made. CHW has completed the ​necessary steps to connect patient to service. Patient may or may not have received service. |
| **Patient Equipped** (i.e., Successful) | Referral has been made. Patient was given all the required information to successfully receive service. Patient has not completed the ​necessary steps to receive service |
| **Failure** (i.e., Not Successful) | Referral has been made. Patient confirmed service was denied or declined. |
| **Disconnected/ Lost to Follow-Up** (i.e., Not Successful) | Referral may or may not have been made. Patient could not be reached on three consecutive attempts (either because the phone number was disconnected, or the patient never answered). |
| **Pending** | Referral has not been made. CHW has not completed the ​necessary steps for patient to receive service. |
| **Secondary Effectiveness Measure:** proportion of patients connected to at least one social service (i.e., received) who resolved or made progress on at least one HRSN | |
| **Resolved** (i.e., Successful) | Patient reported that social need was resolved. Patient no longer needs assistance with social need. |
| **Progress Made** (i.e., Successful) | Patient reported that social need improved but was not fully resolved. |
| **No Progress** (i.e., Not Successful) | Patient feels that they did not receive the help they needed and that they've made no progress regarding the social need they were seeking help with. |
| **Disconnected/ Lost to Follow-Up** (i.e., Not Successful) | Patient confirmed service was received but could not be reached on three consecutive attempts to confirm progress on social need (either because the phone number was disconnected, or the patient never answered). |
